# Retraction of published article

**DOI:** 10.4314/gmj.v57i3.15

**Published:** 2023-09

**Authors:** William Kudzi

Dear Editor-in-Chief,

We would like to retract a published article titled “Hypertension and associated factors among patients attending HIV clinic at Korle-Bu Teaching Hospital” by Edmund T. Nartey, Raymond A. Tetteh, Francis Anto, Bismark Sarfo, William Kudzi and Richard M. Adanu in the March 2023 issue of the *Ghana Medical Journal*.^1^

Thank you for drawing our attention to a closely identical article published earlier in the *Journal of AIDS and Clinical Research, Volume 12: issue 9*, titled “Hypertension and Associated Factors among Patients on HIV Antiretroviral Therapy at Korle-Bu Teaching Hospital” on the 16th of September 2021.^2^

We would like to retract the article from the *Ghana Medical Journal* since the Editorial Board deemed this a duplicate publication.

We do apologise for any inconvenience this duplicate publication has caused.
